# Study of the Magnetized Hybrid Nanofluid Flow through a Flat Elastic Surface with Applications in Solar Energy

**DOI:** 10.3390/ma15217507

**Published:** 2022-10-26

**Authors:** Muhammad Mubashir Bhatti, Hakan F. Öztop, Rahmat Ellahi

**Affiliations:** 1 College of Mathematics and Systems Science, Shandong University of Science and Technology, Qingdao 266590, China; 2 Department of Mechanical Engineering, Technology Faculty, Fırat University, Elazig 23119, Turkey; 3 Center for Modeling & Computer Simulation, Research Institute, King Fahd University of Petroleum & Minerals, Dhahran 31261, Saudi Arabia; 4 Fulbright Fellow Department of Mechanical Engineering, University of California Riverside, Riverside, CA 92521, USA

**Keywords:** hybrid nanofluid, plane elastic surface, viscous dissipation, Brownian motion, magnetic field, numerical simulation, solar energy

## Abstract

The main theme of the present study is to analyze numerically the effects of the magnetic field on the hybrid nanofluid flow over a flat elastic surface. The effects of the thermal and velocity slips are also analyzed in view of the hybrid nanofluid flow. It is considered a combination of titanium oxide (TiO_2_) and copper oxide (CuO) nanoparticles that are suspended in the incompressible and electrically conducting fluid (water). The behavior of the Brownian motion of the nanoparticles and the thermophoretic forces are contemplated in the physical and mathematical formulations. Moreover, the impact of the Joule heating and viscous dissipation are also discussed using the energy equation. The mathematical modeling is simulated with the help of similarity variables. The resulting equations are solved using the Keller–Box method with a combination of finite difference schemes (FDSs). Hybrid nanofluids provide significant advantages over the usual heat transfer fluids. Therefore, the use of nanofluids is beneficial to improve the thermophysical properties of the working fluid. All of the results are discussed for the various physical parameters involved in governing the flow. From the graphical results, it is found that the hybrid nanoparticles improve the concentration, temperature, and velocity profiles, as well as the thickness of the relevant boundary layer. The conjunction of a magnetic field and the velocity slip, strongly opposes the fluid motion. The boundary layer thickness and concentration profile are significantly reduced with the higher levels of the Schmidt number.

## 1. Introduction

The application of nanofluids in heat transfer systems play a critical role in many industrial engineering processes involving chemical and thermal operations. Various fluids have been utilized as heat porters in various heat transfer systems. Heat transfer fluids are beneficial to a variety of applications, including automobile dispensing systems [1,2], power plant heat transfer [3,4], temperature changing systems [5], dispenser mechanisms [6], and renewable energy technology [7]. The thermal conductivity of the heat transfer fluids has a significant influence on the heat transfer process performance and the overall performance of the device. Heat transfer may be accomplished by employing nanofluids. Nanofluids are created by suspending microscopic particles (metallic or non-metallic) in base fluids (such as air, water, silicon fluids, minerals, or aromatic hydrocarbon fluids, propylene glycol/water composites, and synthetic refrigerants). However, when two or more kinds of nanoparticles are present in the base-fluid, the nanofluids are converted into hybrid nanofluids. When they are compared to mono nanofluids, hybrid nanofluids are exceptional and demonstrate a favorable performance [8]. Hybrid nanofluids are the most current and widely employed technology to optimize the heat transfer efficiency [9].

In recent years, the rapid expansion in the world’s population and industrial sectors has contributed to a worldwide energy problem. The use of fossil fuels has increased dramatically, yet their availability is dwindling by the day. Bio-renewable energy, derived from sustainable energy resources, such as solar, geothermal, and wind, is now a viable alternative to fossil fuels. Solar energy is regarded as a vital component of renewable energy for producing electricity and heat. To build solar energy systems, it is critical to achieve a higher heat transfer rate for the improved system performance. As a result, nanofluids are a handy and efficient solution to filling this gap, as well as good for increased heat transmission. There are several benefits for employing nanofluids in solar energy, for instance [10,11,12]:

▪ Nanofluids have remarkable optical characteristics, demonstrating a strong absorption and la ow remittance in both the solar and infrared spectra.
▪ Nanofluids have a greater stability rate and an excellent absorption medium across a broad range of temperatures.
▪ Because of their larger surface area and compact structure, nanoparticles have a considerable influence on the absorption and heat capacity of nanofluids for solar energy systems.
▪ The inclusion of nanofluids in thermal devices minimizes the area of heat transmission, leading to the cost effectiveness of solar energy systems.
▪ In comparison to base fluids, nanofluids substantially optimize the heat conductivity.
▪ The nanoparticles suspended in the host fluids, assist in preventing sedimentation, impediment, and pump and pipe fouling. The nanofluids are an excellent choice for solar energy applications because of this attribute.
▪ The energy efficiency of thermal systems can be improved with nanofluids, which have a greater density and an improved heat transfer coefficient, due to the lower specific heat of the nanoparticles.

Furthermore, solar energy applications benefit greatly from the use of magnetic nanoparticles in the area of heat transfer. The magnetic nanofluids were mostly applied to the fields of electronics, mechanics, material sciences, hydraulics, and solar energy [13,14]. It is known that magnetic nanoparticles function very well in solar cells, capturing solar energy and converting it into electrical energy more effectively than non-magnetic solar cells [15]. Due to the significance of hybrid nanofluids, several authors have studied them, employing different types of nanoparticles that propagate across the two- and three-dimensional structures. 

Tayebi and Chamkha [16] evaluated a buoyancy-driven flow to assess the heat transfer augmentation of the hybrid nanofluid flow over a wavy enclosure. Ghadikolaei et al. [17] addressed the thermophysical characteristics of titanium dioxide nanoparticles with a mixture of copper nanoparticles, and offered a comprehensive study on the shape factor with magnetic implications. Hussein [18] attempted to predict the performance and physical attributes of the hybrid nanofluid flow, employing laminar phenomena through a twin pipe heat exchanger. They discovered that, compared to the base fluid, the hybrid nanofluid significantly improves the thermal efficiency in the heat exchanger. Rostami et al. [19] suggested a mathematical model of the hybrid nanofluid with dual solutions, and analyzed the stagnation flow phenomenon. Ashorynejad and Shahriari [20] developed a magnetic open cavity configuration filled with a hybrid nanofluid, and conducted a thorough examination of the thermophysical behavior. According to their findings, the Nusselt number was drastically lowered, owing to the strong influence of the magnetic field, while the reverse trend was seen for the greater Rayleigh numbers and the nanoparticle volume percentage. Verma et al. [21] examined the efficacy of hybrid nanofluids in flat plate solar collectors, utilizing an innovative host fluid. Aghaei et al. [22] reported a turbulent hybrid nanofluid flow with entropy formation in a trapezoidal enclosure using a magnetic field. They discovered that when the magnetic field is large and the Rayleigh number is low, the Nusselt number has a very perceptible impact. However, the Rayleigh number, the nanoparticle volume percentage, and the magnetic field all contribute to the increased entropy formation. Maskeen et al. [23] evaluated the heat transfer enhancement using copper-alumina nanoparticles embedded in a water-based hybrid nanofluid, swimming in an elastic stretching cylinder. Tayebi and Chamkha [24] researched the natural convection on a hybrid nanofluid travelling through a square chamber with corrugated sidewalls, and provided a comprehensive study of the magnetized entropy formation. They discovered that the conductivity ratio and the magnetic field had a profound impact on the thermal and dynamic fields. Chahregh and Dinarvand [25] researched blood flow in an artery with drug delivery applications while titanium oxide and silver nanoparticles were dispersed. Yang et al. [26] examined the flow of water-based hybrid nanofluids across a flexible surface containing a magnetic dipole. Some more studies are given in the references [27,28,29,30,31,32] on the hybrid nanofluid with various geometrical configurations and body forces. 

During the past years, nanotechnology has grabbed the attention of various researchers because of the fascinating performance and multitudinous heat transfer applications. Hybrid nanofluids provide significant advantages over the usual heat transfer fluids. Therefore, the use of nanofluids is beneficial to improve the thermophysical characteristics of the host fluid. The primary objective of this work is to investigate the behavior of the hybrid nanofluids floating on a flat elastic surface. Hybrid nanofluids are useful and exhibit promising outcomes in solar energy systems. Under the suspension of TiO_2_ and CuO nanoparticles, the water-based nanofluid becomes electrically conductive. The consequences of the velocity and the thermal slip with Joule heating and a viscous dissipation combination, are also examined. Slip effects occur at the surface of several nanomaterial production systems, such as conveyer belts. Slip effects are correlated with the molecular movements in fluids closer to the boundary and tend to cause the non-adherence of the coating’s substrates. These factors may have a significant impact on the momentum characteristics, mass, and heat transfer aspects of a coating extrusion. The velocity equation, energy equation, and nanoparticle concentration equations are represented using the similarity variables, and the resulting equations are solved using the Keller–Box technique. A complete study is offered using the graphical findings and tables. To verify the present findings, the Sherwood number and Nusselt number are compared to the previously published results.

## 2. Mathematical Formulation

**Figure 1:**
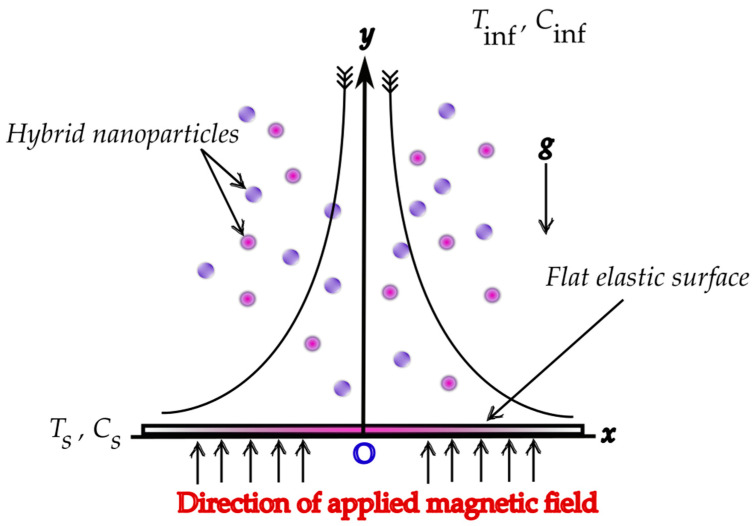
Geometrical structure of the water-based hybrid nanofluid flow over a flat elastic surface with an extrinsic magnetic field.

The electrically conducting water-based hybrid nanofluid flow through an elastic surface is considered. To formulate the physical structure, we have considered the cartesian coordinate system with the *x*-axis assigned along the axial direction, and the *y*-axis assigned along the vertical direction (see Figure 1). The sheet is linearly stretched along the axial direction with a velocity ${\bar{U}}_{s}=ax$, where *a* is the constant and *x* represents the coordinate considered along the elastic surface. A uniform external magnetic field is applied along the *y* direction. We have considered the titanium oxide and copper oxide nanoparticles of spherical shape with a uniform size, suspended in the water-based nanofluid, while the agglomeration is ignored because the hybrid nanofluid is established as a stable composite. The temperature and the concentration at the surface of the elastic plate is assumed to be $T_{s},C_{s}$ while the ambient temperature and concentration is presumed to be $T_{\inf},C_{\inf}$. Furthermore, the velocity and thermal slip are also incorporated in the boundary conditions. In view of the proposed assumptions, the following continuity, momentum, and energy equation are constructed. For instance,

(1) $$\frac{\partial \bar{U}}{\partial x}+\frac{\partial \bar{V}}{\partial y}=0,$$

(2) $$\bar{U}\frac{\partial \bar{U}}{\partial x}+\bar{V}\frac{\partial \bar{U}}{\partial y}=\upsilon_{hnf}\frac{\partial^{2}\bar{U}}{\partial y^{2}}-\frac{\Omega_{hnf}}{\rho_{hnf}}B_{0}^{2}\bar{U},$$

(3) $${\left(\rho c\right)}_{hnf}\left(\bar{U}\frac{\partial \bar{T}}{\partial x}+\bar{V}\frac{\partial \bar{T}}{\partial y}\right)=k_{hnf}\frac{\partial^{2}\bar{T}}{\partial y^{2}}+\mu_{hnf}{\left(\frac{\partial \bar{U}}{\partial y}\right)}^{2}+\Omega_{hnf}B_{0}^{2}{\bar{U}}^{2}+{\left(\rho c_{p}\right)}_{f}\left[D_{t}{\left(\frac{\partial \bar{T}}{\partial y}\right)}^{2}+D_{b}\frac{\partial \bar{T}}{\partial y}\frac{\partial \bar{C}}{\partial y}\right],$$

(4) $$\bar{U}\frac{\partial \bar{C}}{\partial x}+\bar{V}\frac{\partial \bar{C}}{\partial y}=D_{b}\frac{\partial^{2}\bar{C}}{\partial y^{2}}+\frac{D_{t}}{T_{\inf}}{\left(\frac{\partial \bar{T}}{\partial y}\right)}^{2}$$

where $\bar{U},\bar{V}$ indicates the velocity components, υ indicates the kinematic viscosity, Ω is the electrical conductivity, ρ is the density, $B_{0}$ is the applied magnetic field, c is the specific heat of the nanofluid, $c_{p}$ is the specific heat of the nanoparticles, $D_{t}$ is the Brownian diffusion coefficient, $D_{b}$ is the thermophoresis coefficient, k is the thermal conductivity, and hnf represents the hybrid nanofluids. 

### Boundary Conditions with the Slip Effects

The following are the boundary conditions with the slip effects:

(5) $${\bar{U}={\bar{U}}_{s}+v_{slip}\frac{\partial \bar{U}}{\partial y},\bar{V}=0,\bar{T}=T_{s}+T_{slip}\frac{\partial \bar{T}}{\partial y},\bar{C}=C_{s}|}_{y=0},$$

(6) $${\bar{U}\to 0,\bar{V}\to 0,\bar{T}\to T_{\inf},\bar{C}\to C_{\inf}|}_{y=\infty },$$

where $v_{slip}$ is the velocity slip, and $T_{slip}$ is the thermal slip. 

## 3. Similarity Analysis

The similarity variables are introduced as follows:

(7) $$\eta =y\sqrt{\frac{a}{\upsilon_{f}}},\bar{U}={\bar{U}}_{w}f^{\prime },\bar{V}=-\sqrt{\upsilon_{f}a}f,\theta =\frac{\bar{T}-T_{\inf}}{T_{s}-T_{\inf}},\varphi =\frac{C-C_{\inf}}{C_{s}-C_{\inf}}.$$

Employing Equation (7) in the governing equations, we obtain the following set of nonlinear differential equations:

(8) $$\frac{E_{1}}{E_{2}}f^{\prime \prime \prime }+ff^{\prime \prime }-{f^{\prime }}^{2}-\frac{E_{3}}{E_{2}}\beta f^{\prime }=0,$$

(9) $$\frac{E_{4}}{\chi }\theta^{\prime \prime }+E_{5}f\theta^{\prime }+\omega_{t}\theta^{\prime }\varphi^{\prime }+\omega_{b}{\theta^{\prime }}^{2}+\lambda \left(E_{1}{f^{\prime \prime }}^{2}+E_{3}\beta {f^{\prime }}^{2}\right)=0,$$

(10) $$\varphi^{\prime \prime }+\Lambda f\varphi^{\prime }+\frac{\omega_{t}}{\omega_{b}}\theta^{\prime \prime }=0,$$

The boundary conditions are

(11) $$f^{\prime }-1=\delta_{1}f^{\prime \prime }\left(0\right),f=0,\theta -1=\delta_{2}\theta^{\prime }\left(0\right),\varphi =1,\;\mathrm{at}\;\eta =0,$$

(12) $$\underset{\eta \to 0}{\lim}f^{\prime }\to 0,\underset{\eta \to 0}{\lim}\theta \to 0,\underset{\eta \to 0}{\lim}\varphi \to 0.$$

where β is the magnetic parameter, χ is the Prandtl number, $\omega_{b}$ is the Brownian motion parameter, $\omega_{t}$ is the thermophoresis parameter, λ is the Eckert number, $\delta_{1}$ is the dimensionless velocity slip, $\delta_{2}$ is the thermal slip, and Λ is the Schmidt number. These parameters are defined as:

(13) $$\begin{array}{l} \chi =\frac{\upsilon_{f}}{\bar{\alpha }},\Lambda =\frac{\upsilon_{f}}{D_{b}},\bar{\alpha }=\frac{k_{f}}{{\left(\rho c\right)}_{f}},\beta =\frac{\Omega_{f}B_{0}^{2}}{a\rho_{f}},\delta_{1}=v_{slip}\sqrt{\frac{a}{\upsilon_{f}}},\delta_{2}=T_{slip}\sqrt{\frac{a}{\upsilon_{f}}},\\ \lambda =\frac{{\bar{U}}_{s}^{2}}{c_{f}\left(T_{s}-T_{\inf}\right)},\omega_{b}=\frac{\Gamma D_{b}\left(C_{s}-C_{\inf}\right)}{\upsilon_{f}},\omega_{t}=\frac{\Gamma D_{t}\left(T_{s}-T_{\inf}\right)}{\upsilon_{f}T_{\inf}},\Gamma =\frac{{\left(\rho c_{p}\right)}_{f}}{{\left(\rho c\right)}_{f}}, \end{array}|$$

and

(14) $$E_{1}=\frac{\mu_{hnf}}{\mu_{f}},E_{2}=\frac{\rho_{hnf}}{\rho_{f}},E_{3}=\frac{\Omega_{hnf}}{\Omega_{f}},E_{4}=\frac{k_{hnf}}{k_{f}},E_{5}=\frac{{\left(\rho c\right)}_{hnf}}{{\left(\rho c\right)}_{f}}.$$

The mathematical expressions of $E_{m}\left(m=1-5\right)$, are provided in Table 1. 

## 4. Finite Difference and the Keller–Box Methods 

Since there is no way to solve Equations (9)–(11) precisely, we will use the Keller–Box approach, in conjunction with Newton’s method, and the finite difference method. The main steps for the proposed methodology are described below [33]:

i. Convert the obtained differential equations to the first order differential equations.
ii. Using the finite difference approach, transform the reduced differential equation.
iii. Using Newton’s approach, convert the resultant nonlinear algebraic equations to the linearized algebraic equations.
iv. Utilize the block tri-diagonal elimination strategy to solve the formulated equations.

### 4.1. Finite Difference Approach

To apply the suggested approach to the following Equations (9)–(11), we must first reduce these differential equations to the first order differential equations, we obtain:

(15) $$f^{\prime }=P,P^{\prime }=Q,\theta^{\prime }=T,\varphi^{\prime }=V,$$

Then

(16) $$\begin{array}{l} \frac{E_{1}}{E_{2}}Q^{\prime }+fQ-P^{2}-\frac{E_{3}}{E_{2}}\beta P=0,\\ \frac{E_{4}}{\chi }T^{\prime }+E_{5}fT+\omega_{b}\times TV+\omega_{t}\times T^{2}+\lambda \left(E_{1}Q^{2}+E_{3}\beta P^{2}\right)=0,\\ V^{\prime }+\Lambda fV+\frac{\omega_{t}}{\omega_{b}}T^{\prime }=0. \end{array}|$$

The following equations will be used to express the boundary conditions:

(17) $$\begin{array}{l} P\left(0\right)=1+\delta_{1}Q\left(0\right),\varphi \left(0\right)=1,f\left(0\right)=0,\theta \left(0\right)=1+\delta_{2}T\left(0\right),\\ P\left(\infty \right)\to 0,\theta \left(\infty \right)\to 0,\varphi \left(\infty \right)\to 0. \end{array}|$$

Now, let us examine the $\eta_{j-1}\eta_{j}$ segment with $\eta_{j-1/2}$ as the midpoint, which is written as follows:

(18) $$\eta_{0}=0,\eta_{j}=\eta_{j-1}+h_{j},\eta_{j}=\eta_{\infty },$$

where $h_{j}$ is the Δη− spacing and Δη=1,2,…J, is a sequence number that indicates the coordinate location. The finite difference approximations for the mid-point $\eta_{j-1/2}$ are described as:

(19) $$\begin{array}{l} \frac{f_{j}-f_{j-1}}{h_{j}}=\frac{P_{j}+P_{j-1}}{2},\\ \frac{P_{j}-P_{j-1}}{h_{j}}=\frac{Q_{j}+Q_{j-1}}{2},\\ \frac{\theta_{j}-\theta_{j-1}}{h_{j}}=\frac{T_{j}+T_{j-1}}{2},\\ \frac{\varphi_{j}-\varphi_{j-1}}{h_{j}}=\frac{V_{j}+V_{j-1}}{2},\\ \frac{E_{1}}{E_{2}}\frac{Q_{j}-Q_{j-1}}{h_{j}}+{\left(fQ\right)}_{j-1/2}-{\left(P_{j-1/2}\right)}^{2}-\frac{E_{3}}{E_{2}}\beta P_{j-1/2}=0,\\ \frac{E_{4}}{\chi }\frac{T_{j}-T_{j-1}}{h_{j}}+E_{5}{\left(fT\right)}_{j-1/2}+\omega_{b}{\left(TV\right)}_{j-1/2}+\omega_{t}{\left(T_{j-1/2}\right)}^{2}+\lambda \left[E_{1}{\left(Q_{j-1/2}\right)}^{2}+E_{3}\beta {\left(P_{j-1/2}\right)}^{2}\right]=0,\\ \frac{V_{j}-V_{j-1}}{h_{j}}+\Lambda {\left(fV\right)}_{j-1/2}+\left(\frac{\omega_{t}}{\omega_{b}}\right)\frac{T_{j}-T_{j-1}}{h_{j}}=0. \end{array}|$$

Equation (19) is used for j=1,2,…J, and the modified boundary layer thickness $\eta_{j}$ must be big enough to expand beyond the boundary layer. The boundary conditions are as follows:

(20) $$\begin{array}{l} P_{0}\left(0\right)=1+\delta_{1}Q_{0}\left(0\right),\varphi_{0}\left(0\right)=1,f_{0}\left(0\right)=0,\theta_{0}\left(0\right)=1+\delta_{2}T_{0}\left(0\right),\\ P_{J}\left(\infty \right)\to 0,\theta_{J}\left(\infty \right)\to 0,\varphi_{J}\left(\infty \right)\to 0. \end{array}|$$

### 4.2. Newton’s Method

The aforementioned Equations (19)–(20) are algebraic nonlinear equations; consequently, Newton’s technique will be used to make them linear. Let us write the Newton iterates, as follows, for the (k+1)th iterates, we write:

(21) $$\begin{array}{l} f_{j}^{k+1}=f_{j}^{k}+\Delta f_{j}^{k},\\ P_{j}^{k+1}=P_{j}^{k}+\Delta P_{j}^{k},\\ Q_{j}^{k+1}=Q_{j}^{k}+\Delta Q_{j}^{k},\\ \theta_{j}^{k+1}=\theta_{j}^{k}+\Delta \theta_{j}^{k},\\ T_{j}^{k+1}=T_{j}^{k}+\Delta T_{j}^{k},\\ \varphi_{j}^{k+1}=\varphi_{j}^{k}+\Delta \varphi_{j}^{k},\\ V_{j}^{k+1}=V_{j}^{k}+\Delta V_{j}^{k}, \end{array}|$$

where k=0,1,2,….

Applying the aforementioned equation to the formulated equations, yields

(22) $$\begin{array}{l} \frac{\Delta f_{j}-\Delta f_{j-1}}{h_{j}}-\Delta \left(\frac{P_{j}+P_{j-1}}{2}\right)={\left(R_{1}\right)}_{j-1/2},\\ \frac{\Delta P_{j}-\Delta P_{j-1}}{h_{j}}-\Delta \left(\frac{Q_{j}+Q_{j-1}}{2}\right)={\left(R_{2}\right)}_{j-1/2},\\ \frac{\Delta \theta_{j}-\Delta \theta_{j-1}}{h_{j}}-\Delta \left(\frac{T_{j}+T_{j-1}}{2}\right)={\left(R_{3}\right)}_{j-1/2},\\ \frac{\Delta \varphi_{j}-\Delta \varphi_{j-1}}{h_{j}}-\Delta \left(\frac{V_{j}+V_{j-1}}{2}\right)={\left(R_{4}\right)}_{j-1/2}, \end{array}|$$

(23) $$\begin{array}{l} \left(a_{1}\right)\Delta Q_{j}+\left(a_{2}\right)\Delta Q_{j-1}+\left(a_{3}\right)\Delta f_{j}+\left(a_{4}\right)\Delta f_{j-1}+\left(a_{5}\right)\Delta P_{j}+\left(a_{6}\right)\Delta P_{j-1}={\left(R_{5}\right)}_{j-1/2},\\ \left[\begin{array}{l} \left(b_{1}\right)\Delta T_{j}+\left(b_{2}\right)\Delta T_{j-1}+\left(b_{3}\right)\Delta f_{j}+\left(b_{4}\right)\Delta f_{j-1}\\ +\left(b_{5}\right)\Delta V_{j}+\left(b_{6}\right)\Delta V_{j-1}+\left(b_{7}\right)\Delta Q_{j}+\left(b_{8}\right)\Delta Q_{j-1}\\ +\left(b_{9}\right)\Delta P_{j}+\left(b_{10}\right)\Delta P_{j-1} \end{array}\right]={\left(R_{6}\right)}_{j-1/2},\\ \left[\left(c_{1}\right)\Delta V_{j}+\left(c_{2}\right)\Delta V_{j-1}+\left(c_{3}\right)\Delta f_{j}+\left(c_{4}\right)\Delta f_{j-1}+\left(c_{5}\right)\Delta T_{j}+\left(c_{6}\right)\Delta T_{j-1}\right]={\left(R_{7}\right)}_{j-1/2}, \end{array}|$$

and

(24) $$\begin{array}{l} {\left(a_{1}\right)}_{j}=\frac{E_{1}}{E_{2}}+\frac{h_{j}}{2}f_{j-1/2},{\left(a_{2}\right)}_{j}={\left(a_{1}\right)}_{j}-2\frac{E_{1}}{E_{2}},\\ {\left(a_{3}\right)}_{j}=\frac{h_{j}}{2}Q_{j-1/2},{\left(a_{4}\right)}_{j}={\left(a_{3}\right)}_{j},\\ {\left(a_{5}\right)}_{j}=-h_{j}P_{j-1/2}-\frac{\beta }{2}\frac{E_{3}}{E_{2}}h_{j},{\left(a_{6}\right)}_{j}={\left(a_{5}\right)}_{j} \end{array}|$$

(25) $$\begin{array}{l} {\left(b_{1}\right)}_{j}=\frac{E_{4}}{\chi }+E_{5}\frac{h_{j}}{2}f_{j-1/2}+\omega_{b}\frac{h_{j}}{2}V_{j-1/2}+\omega_{t}h_{j}T_{j-1/2},{\left(b_{2}\right)}_{j}={\left(b_{1}\right)}_{j}-\frac{2}{\chi }E_{4},\\ {\left(b_{3}\right)}_{j}=E_{5}\frac{h_{j}}{2}T_{j-1/2},{\left(b_{4}\right)}_{j}={\left(b_{3}\right)}_{j},{\left(b_{5}\right)}_{j}=\omega_{b}\frac{h_{j}}{2}T_{j-1/2},\\ {\left(b_{6}\right)}_{j}={\left(b_{5}\right)}_{j},{\left(b_{7}\right)}_{j}=E_{1}\lambda \cdot \frac{h_{j}}{2}Q_{j-1/2},{\left(b_{8}\right)}_{j}={\left(b_{7}\right)}_{j},\\ {\left(b_{9}\right)}_{j}=\lambda \cdot \frac{h_{j}}{2}E_{3}\beta P_{j-1/2},{\left(b_{10}\right)}_{j}={\left(b_{9}\right)}_{j}, \end{array}|$$

(26) $$\begin{array}{l} {\left(c_{1}\right)}_{j}=1+\frac{h_{j}}{2}\Lambda f_{j-1/2},{\left(c_{2}\right)}_{j}={\left(c_{1}\right)}_{j}-2,{\left(c_{3}\right)}_{j}=\Lambda \cdot \frac{h_{j}}{2}V_{j-1/2},\\ {\left(c_{4}\right)}_{j}={\left(c_{3}\right)}_{j},{\left(c_{5}\right)}_{j}=\frac{\omega_{t}}{\omega_{b}},{\left(c_{6}\right)}_{j}=-{\left(c_{5}\right)}_{j}, \end{array}|$$

(27) $$\begin{array}{l} \left(R_{1}\right)=\frac{f_{j-1}-f_{j}}{h_{j}}+\frac{P_{j}+P_{j-1}}{2},\\ \left(R_{2}\right)=\frac{P_{j-1}-P_{j}}{h_{j}}+\frac{Q_{j}+Q_{j-1}}{2},\\ \left(R_{3}\right)=\frac{\theta_{j-1}-\theta_{j}}{h_{j}}+\frac{T_{j}+T_{j-1}}{2},\\ \left(R_{4}\right)=\frac{\varphi_{j-1}-\varphi_{j}}{h_{j}}+\frac{V_{j}+V_{j-1}}{2},\\ \left(R_{5}\right)=\frac{E_{1}}{E_{2}}\frac{Q_{j-1}-Q_{j}}{h_{j}}-{\left(hQ\right)}_{j-1/2}+{\left(P_{j-1/2}\right)}^{2}+\frac{E_{3}}{E_{2}}\beta P_{j-1/2},\\ \left(R_{6}\right)=\frac{E_{4}}{\chi }\frac{T_{j-1}-T_{j}}{h_{j}}-E_{5}{\left(fT\right)}_{j-1/2}-\omega_{b}{\left(TV\right)}_{j-1/2}\\ -\omega_{t}{\left(T_{j-1/2}\right)}^{2}-\lambda \left[E_{1}{\left(Q_{j-1/2}\right)}^{2}+E_{3}\beta {\left(P_{j-1/2}\right)}^{2}\right],\\ \left(R_{7}\right)=\frac{V_{j-1}-V_{j}}{h_{j}}-\Lambda {\left(fV\right)}_{j-1/2}-\left(\frac{\omega_{t}}{\omega_{b}}\right)\frac{T_{j}-T_{j-1}}{h_{j}}=0 \end{array}|$$

The boundary conditions become

(28) $$\begin{array}{l} \Delta f_{0}\left(0\right)=0,\Delta P_{0}=\delta_{1}\Delta Q_{0},\Delta \theta_{0}\left(0\right)=\delta_{2}\Delta T_{0},\Delta \varphi_{0}\left(0\right)=0,\\ \Delta P_{j}\left(\infty \right)\to 0,\Delta \theta_{j}\left(\infty \right)\to 0,\Delta \varphi_{j}\left(\infty \right)\to 0 \end{array}|$$

### 4.3. Block Elimination Method

Since the system has a block-tridiagonal structure, it is possible to solve the linearized differential Equations (22)–(28) using the block elimination technique. This technique was developed by Cebeci and Bradshaw [34]. The block-tridiagonal structure often comprises constants or variables; however, we can see here that it is composed of block matrices. The matrix form of Equations (22)–(28) is given as:

(29) AΔ=r

and

(30) $$A=\left[\begin{array}{l} \begin{array}{ccc} \left[A_{1}\right] & \left[C_{1}\right] & \\ \left[B_{2}\right] & \left[A_{2}\right] & \left[C_{2}\right]\\ & \ddots & \ddots \end{array}\begin{array}{ccc} \ddots & & \end{array}\\ \begin{array}{ccc} \ddots & \ddots & \\ \left[B_{j-1}\right] & \left[A_{j-1}\right] & \left[C_{j-1}\right]\\ & \left[B_{j}\right] & \left[A_{j}\right] \end{array} \end{array}\right],\Delta =\left[\begin{array}{l} \left[\Delta_{1}\right]\\ \left[\Delta_{2}\right]\\ \left[\Delta_{3}\right]\\ \vdots \\ \left[\Delta_{j-1}\right]\\ \left[\Delta_{j}\right] \end{array}\right],r=\left[\begin{array}{l} \left[R_{1}\right]\\ \left[R_{2}\right]\\ \left[R_{3}\right]\\ \vdots \\ \left[R_{j-1}\right]\\ \left[R_{j}\right] \end{array}\right],$$

The matrices’ elements are identified as follows:

(31) $$\left[A_{1}\right]=\left[\begin{array}{ccccccc} 0 & 0 & 0 & 1 & 0 & 0 & 0\\ -\frac{H_{j}}{2} & 0 & 0 & 0 & -\frac{H_{j}}{2} & 0 & 0\\ 0 & -\frac{H_{j}}{2} & 0 & 0 & 0 & -\frac{H_{j}}{2} & 0\\ 0 & 0 & -\frac{H_{j}}{2} & 0 & 0 & 0 & -\frac{H_{j}}{2}\\ {\left(a_{2}\right)}_{1} & 0 & 0 & {\left(a_{3}\right)}_{1} & {\left(a_{1}\right)}_{1} & 0 & 0\\ {\left(b_{8}\right)}_{1} & {\left(b_{2}\right)}_{1} & {\left(b_{6}\right)}_{1} & {\left(b_{3}\right)}_{1} & {\left(b_{7}\right)}_{1} & {\left(b_{1}\right)}_{1} & {\left(b_{5}\right)}_{1}\\ 0 & {\left(c_{6}\right)}_{1} & {\left(c_{2}\right)}_{1} & {\left(c_{3}\right)}_{1} & 0 & {\left(c_{5}\right)}_{1} & {\left(c_{1}\right)}_{1} \end{array}\right]$$

(32) $${\left[A_{j}\right]}_{2\leq j\leq J}=\left[\begin{array}{ccccccc} -\frac{H_{j}}{2} & 0 & 0 & 1 & 0 & 0 & 0\\ -1 & 0 & 0 & 0 & -\frac{H_{j}}{2} & 0 & 0\\ 0 & -1 & 0 & 0 & 0 & -\frac{H_{j}}{2} & 0\\ 0 & 0 & -1 & 0 & 0 & 0 & -\frac{H_{j}}{2}\\ {\left(a_{6}\right)}_{j} & 0 & 0 & {\left(a_{3}\right)}_{j} & {\left(a_{1}\right)}_{j} & 0 & 0\\ {\left(b_{10}\right)}_{j} & 0 & 0 & {\left(b_{3}\right)}_{j} & {\left(b_{7}\right)}_{j} & {\left(b_{1}\right)}_{j} & {\left(b_{5}\right)}_{j}\\ 0 & 0 & 0 & {\left(c_{3}\right)}_{j} & 0 & {\left(c_{5}\right)}_{j} & {\left(c_{1}\right)}_{j} \end{array}\right]$$

(33) $$\left[A_{1}\right]={\left[B_{j}\right]}_{2\leq j\leq J}=\left[\begin{array}{ccccccc} 0 & 0 & 0 & -1 & 0 & 0 & 0\\ 0 & 0 & 0 & 0 & -\frac{H_{j}}{2} & 0 & 0\\ 0 & 0 & 0 & 0 & 0 & -\frac{H_{j}}{2} & 0\\ 0 & 0 & 0 & 0 & 0 & 0 & -\frac{H_{j}}{2}\\ 0 & 0 & 0 & {\left(a_{4}\right)}_{j} & {\left(a_{2}\right)}_{j} & 0 & 0\\ 0 & 0 & 0 & {\left(b_{4}\right)}_{j} & {\left(b_{8}\right)}_{j} & {\left(b_{2}\right)}_{j} & {\left(b_{6}\right)}_{j}\\ 0 & 0 & 0 & {\left(c_{4}\right)}_{j} & 0 & {\left(c_{6}\right)}_{j} & {\left(c_{2}\right)}_{j} \end{array}\right]$$

(34) $${\left[C_{j}\right]}_{1\leq j\leq J-1}=\left[\begin{array}{ccccccc} -\frac{H_{j}}{2} & 0 & 0 & 0 & 0 & 0 & 0\\ 1 & 0 & 0 & 0 & 0 & 0 & 0\\ 0 & 1 & 0 & 0 & 0 & 0 & 0\\ 0 & 0 & 1 & 0 & 0 & 0 & 0\\ {\left(a_{5}\right)}_{j} & 0 & 0 & 0 & 0 & 0 & 0\\ {\left(b_{9}\right)}_{j} & 0 & 0 & 0 & 0 & 0 & 0\\ 0 & 0 & 0 & 0 & 0 & 0 & 0 \end{array}\right]$$

(35) $$\left[\Delta_{1}\right]=\left[\begin{array}{l} \Delta Q_{0}\\ \Delta T_{0}\\ \Delta V_{0}\\ \Delta f_{1}\\ \Delta Q_{1}\\ \Delta T_{1}\\ \Delta V_{1} \end{array}\right],{\left[\Delta_{j}\right]}_{2\leq j\leq J}=\left[\begin{array}{l} \Delta P_{j-1}\\ \Delta \theta_{j-1}\\ \Delta \varphi_{j-1}\\ \Delta f_{j}\\ \Delta Q_{j}\\ \Delta T_{j}\\ \Delta V_{j} \end{array}\right],$$

(36) $${\left[R_{j}\right]}_{1\leq j\leq J}=\left[\begin{array}{l} {\left(R_{1}\right)}_{j-1/2}\\ {\left(R_{2}\right)}_{j-1/2}\\ {\left(R_{3}\right)}_{j-1/2}\\ {\left(R_{4}\right)}_{j-1/2}\\ {\left(R_{5}\right)}_{j-1/2}\\ {\left(R_{6}\right)}_{j-1/2}\\ {\left(R_{7}\right)}_{j-1/2} \end{array}\right],$$

We suppose that A is a nonsingular matrix and it can be factorized as

(37) A=LU

and

(38) $$L=\left[\begin{array}{l} \begin{array}{ccc} \left[\alpha_{1}\right] & \left[C_{1}\right] & \\ \left[B_{2}\right] & \left[\alpha_{2}\right] & \left[C_{2}\right]\\ & \ddots & \ddots \end{array}\begin{array}{ccc} \ddots & & \end{array}\\ \begin{array}{ccc} \ddots & \ddots & \\ \left[B_{j-1}\right] & \left[\alpha_{j-1}\right] & \left[C_{j-1}\right]\\ & \left[B_{j}\right] & \left[\alpha_{j}\right] \end{array} \end{array}\right],$$

(39) $$U=\left[\begin{array}{l} \begin{array}{ccc} \left[I_{1}\right] & \left[\Gamma_{1}\right] & \\ & \left[I_{2}\right] & \left[\Gamma_{2}\right]\\ & \ddots & \ddots \end{array}\begin{array}{ccc} \ddots & & \end{array}\\ \begin{array}{ccc} \ddots & \ddots & \\ & \left[I_{j-1}\right] & \left[\Gamma_{j-1}\right]\\ & & \left[I_{j}\right] \end{array} \end{array}\right],$$

where I is an 7×7 identity matrix, while $\left[\alpha_{i}\right]$ and $\left[\Gamma_{i}\right]$ are 7×7 matrices in which the elements can be obtained using the following equations

(40) $$\begin{array}{l} \left[\alpha_{1}\right]=\left[A_{1}\right],\\ \left[A_{1}\right]\left[\Gamma_{1}\right]=\left[C_{1}\right],\\ {\left[\alpha_{j}\right]}_{j=2,3,\ldots ,J}=\left[A_{j}\right]-\left[B_{j}\right]\left[\Gamma_{j-1}\right],\\ \left[\alpha_{j}\right]{\left[\Gamma_{j}\right]}_{j=2,3,\ldots ,J-1}=\left[C_{j}\right], \end{array}|$$

Substituting the above equations into the Equation (29), we obtain

(41) LUΔ=r,

If we define

(42) UΔ=W,

Then Equation (41) becomes

(43) LW=r,

where

(44) $$W=\left[\begin{array}{l} \left[W_{1}\right]\\ \left[W_{2}\right]\\ \left[W_{3}\right]\\ \vdots \\ \left[W_{j-1}\right]\\ \left[W_{j}\right] \end{array}\right],$$

The following relationships may be used to solve the components in the aforementioned Equation (44):

$$\begin{array}{l} \left[\alpha_{1}\right]\left[W_{1}\right]=\left[R_{1}\right],\\ \left[\alpha_{j}\right]{\left[W_{j}\right]}_{2\leq j\leq J}=\left[R_{j}\right]-\left[B_{j}\right]\left[W_{j-1}\right], \end{array}|$$

When the elements of *W* can be determined using the above equation, the result for Δ may be determined using the following relation:

(45) $$\begin{array}{l} \left[\Delta_{j}\right]=\left[W_{j}\right],\\ {\left[\Delta_{j}\right]}_{1\leq j\leq J-1}=\left[W_{j}\right]-\left[\Gamma_{j}\right]\left[\delta_{j+1}\right], \end{array}|$$

These computations are repeated until a certain convergence criterion is met, at which point the calculations are terminated. This is only feasible if the first guesses are chosen correctly. The first estimate may be selected using the specified boundary conditions. For instance, in the current investigation, we chose the following starting guesses:

(46) $$f_{0}=\frac{1-e^{-\eta }}{1+\delta_{1}},\theta_{0}=\frac{e^{-\eta }}{1+\delta_{2}},\varphi_{0}=e^{-\eta },$$

A uniform grid size is taken Δη=0.006 and is found to satisfy the convergence and the solutions are obtained with an error of tolerance $10^{-5}$. 

## 5. Physical Quantities 

The physical quantities of engineering interest, such as skin friction, the local Nusselt number, and the local Sherwood number are defined as:

(47) $$C_{Fx}=\frac{\mu_{hnf}ℑ}{{\bar{U}}_{s}^{2}\rho },N_{Ux}=\frac{xℵ}{k_{f}\left(T_{s}-T_{\inf}\right)},S_{Hx}=\frac{x{ℜ}_{h}}{\left(T_{s}-T_{\inf}\right)},$$

where ℑ, ℵ, ${ℜ}_{h}$ are wall shear stress, heat flux, and the mass flux. They are defined as:

(48) $$ℑ={\frac{\partial \bar{U}}{\partial y}|}_{y=0},ℵ=-k_{hnf}{\frac{\partial \bar{T}}{\partial y}|}_{y=0},{ℜ}_{h}=-{\frac{\partial \bar{C}}{\partial y}|}_{y=0}.$$

In the dimensionless form, they are written as:

(49) $$\sqrt{ℜe}C_{Fx}=E_{1}f^{\prime \prime }\left(0\right),\frac{N_{Ux}}{\sqrt{ℜe}}=-E_{4}\theta^{\prime }\left(0\right),\frac{S_{Hx}}{\sqrt{ℜe}}=-\varphi^{\prime }\left(0\right),$$

## 6. Discussion of the Graphical and Numerical Results 

In this part, we will examine the graphical and numerical outcomes of all of the key parameters included in the mathematical modeling. We used the following parametric values to do the numerical computations:$\Lambda =10;\;\chi =6.2;\;\omega_{b}=0.1;\;\omega_{t}=0.1;\;\lambda =0.1;\;\delta_{1}=0.1;\;\delta_{2}=0.1;\;\beta =1$. The numerical values of the copper oxide and titanium oxide nanoparticles and water are given in Table 2. We conducted a numerical comparison to corroborate the current findings with the previously published results by Khan and Pop [35], as shown in Table 3. In the absence of nanoparticles, the magnetic field and slip effects, a numerical comparison of the Nusselt number and the Sherwood number profiles was carried out. The comparison demonstrates that the current findings are in great agreement, and that the current results for the hybrid nanofluids are valid. This also demonstrates that the current findings converge for the specified parametric parameters. The skin friction profile, the Sherwood number and the Nusselt number profile are computed and presented in Table 4.

### 6.1. Velocity Curves

**Figure 2:**
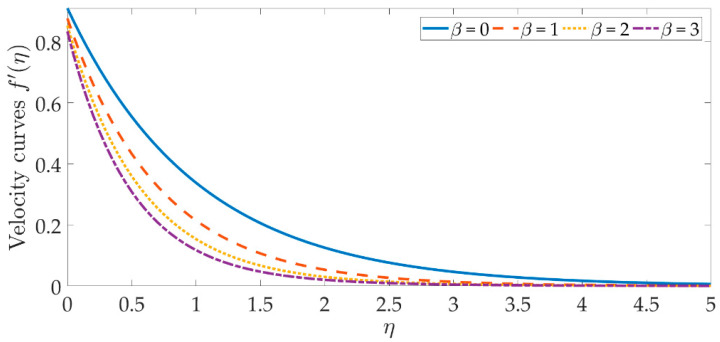
Velocity curves against the multiple values of β.

**Figure 3:**
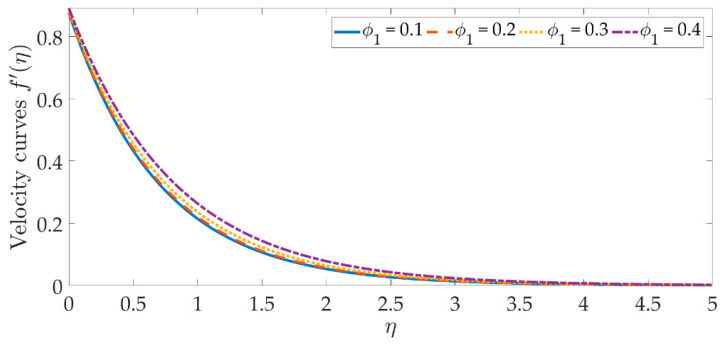
Velocity curves against the multiple values of $\phi_{1}$.

**Figure 4:**
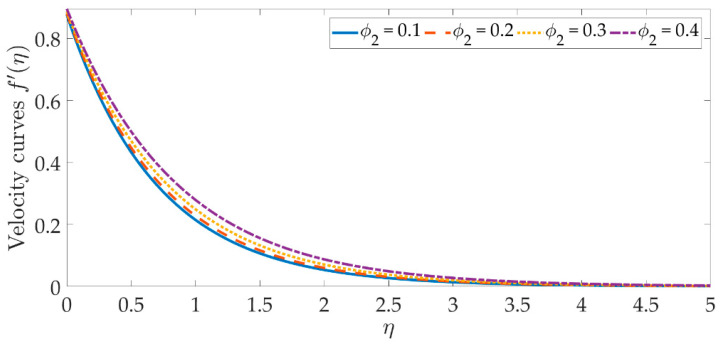
Velocity curves against the multiple values of $\phi_{2}$.

**Figure 5:**
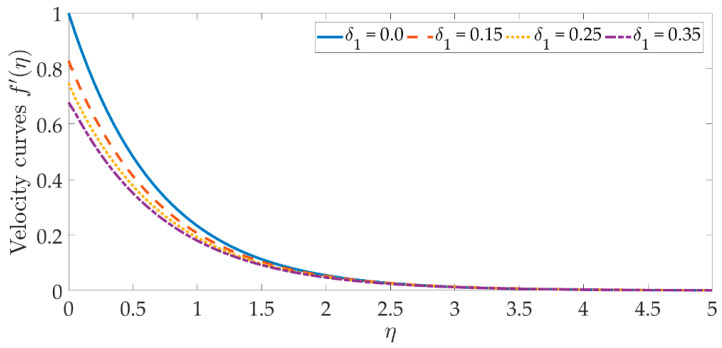
Velocity curves against the multiple values of $\delta_{1}$.

To see the graphical mechanism of the velocity profile versus the various parameters, Figure 2, Figure 3, Figure 4 and Figure 5 can be presented. The flow behavior under the influence of a magnetic field is shown in Figure 2. In this case, β=0 represents a magnetic field that is entirely absent. As we can see, the velocity profile and the boundary layer thickness significantly decrease as the magnetic field’s intensity rises. The resistive Lorentz force is produced when a magnetic field is present, which lowers the flow velocity. The magnetic field’s existence helps to regulate the velocity of the fluid. The significant flow control may be accomplished with the strategic selection of the magnetic fields in various industrial engineering, such as the processing of magnetic nanomaterials, allowing engineers to make internal modifications to the nano-polymers. Figure 3 and Figure 4 indicate that increasing the volume percentage of the nanoparticles of copper oxide and titanium oxide $\phi_{1},\phi_{2}$ strengthens the velocity field. However, with smaller values of the nanoparticle concentration, a weaker velocity field is formed. The velocity profile is dramatically depleted by the slip parameter $\delta_{1}$, as seen in Figure 5. As a consequence of the non-adherence of the nanofluid closer to the surface, the momentum diffusivity is hindered in this area. It’s worth noting that when the hydrodynamics slip is disregarded, the amplitude of the velocity field is significantly overestimated. To obtain more accurate predictions of the dynamics of a stretched surface, scientists and engineers should incorporate the slide effects into the mathematical models. 

### 6.2. Temperature Profile

**Figure 6:**
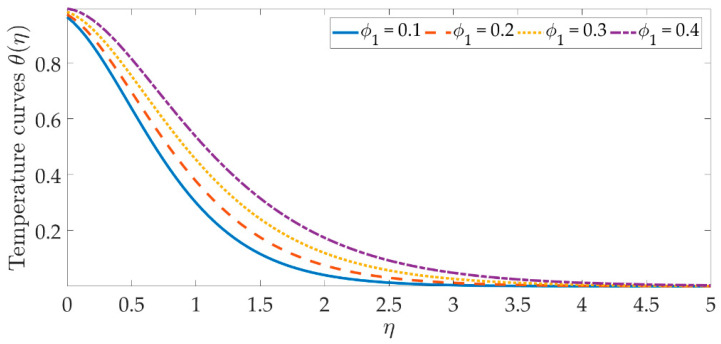
Temperature curves against the multiple values of $\phi_{1}$.

**Figure 7:**
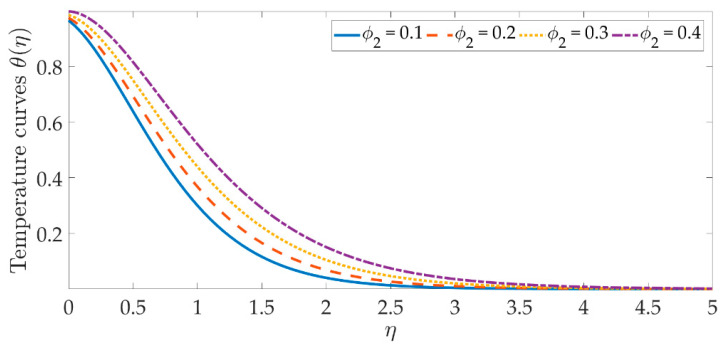
Temperature curves against the multiple values of $\phi_{2}$.

**Figure 8:**
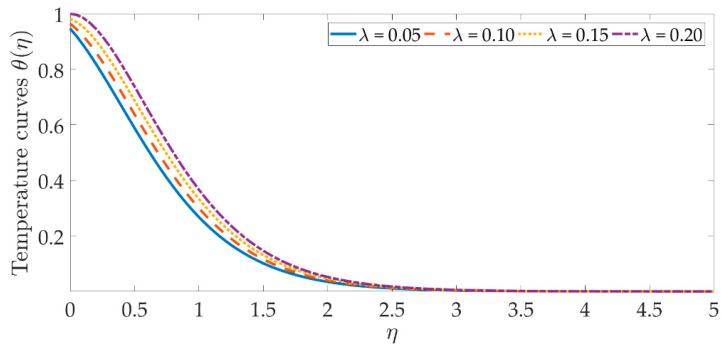
Temperature curves against the multiple values of λ.

**Figure 9:**
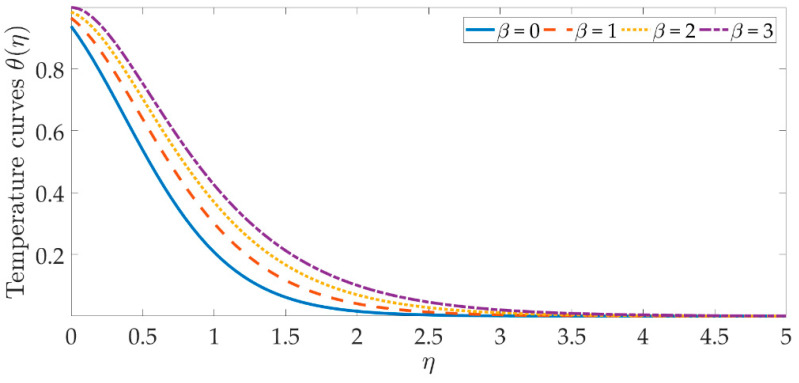
Temperature curves against the multiple values of β.

**Figure 10:**
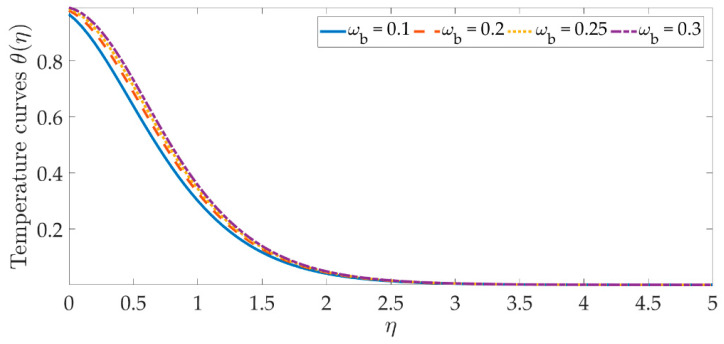
Temperature curves against the multiple values of $\omega_{b}$.

**Figure 11:**
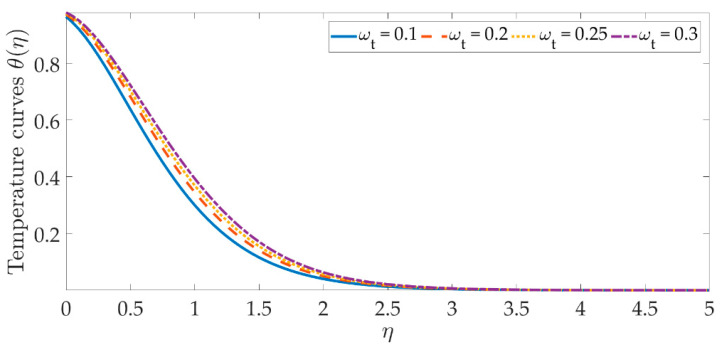
Temperature curves against the multiple values of $\omega_{t}$.

**Figure 12:**
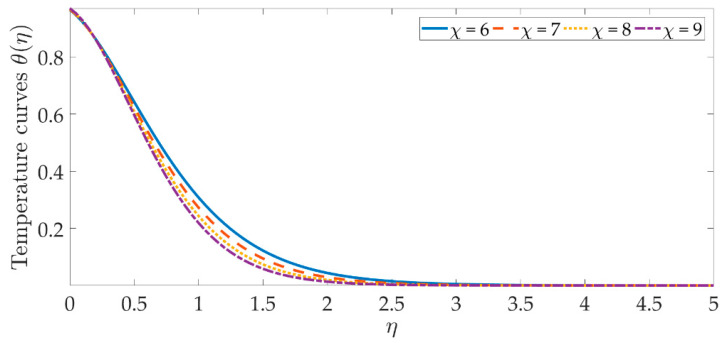
Temperature curves against the multiple values of χ.

**Figure 13:**
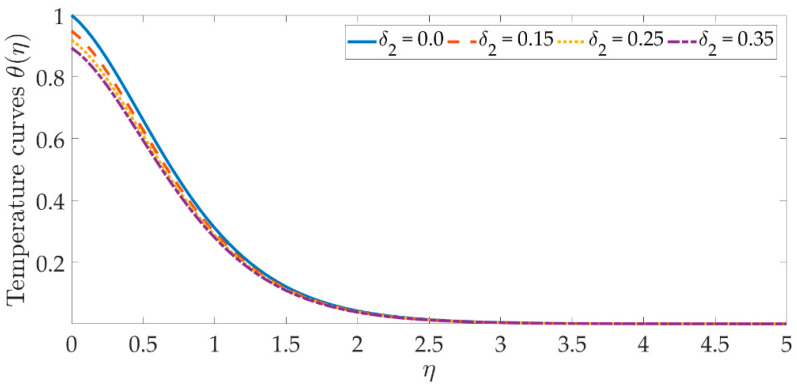
Temperature curves against the multiple values of $\delta_{2}$.

The temperature profile behavior is shown in Figure 6, Figure 7, Figure 8, Figure 9, Figure 10, Figure 11, Figure 12 and Figure 13, for the various values of all of the emerging parameters. The variations of the nanoparticle volume fractions $\phi_{1},\phi_{2}$ on the temperature profile is seen in Figure 6 and Figure 7. We can observe that the thickness of the boundary layer and the thermal profile are both evenly improved by the presence of the nanoparticles. Therefore, the increased doping with the nanoparticles (i.e., CuO and TiO_2_), displays a beneficial improvement in the thermal profile, especially towards the wall. Figure 8 shows that the Eckert number increased the thermal profile across the entire domain. Since the Eckert number is directly proportional to the advective mass transfer (see Equation (13)), enhancing the Eckert number boosts the advective mass, resulting in a boost in the thermal profile. Figure 9 depicts the impact of the magnetic parameter’s fluctuation on the temperature profile. As the magnetic field’s values rise, it is seen that the temperature profile along the surface is elevated. Such effects result from the inclusion of $+\lambda \beta E_{3}{f^{\prime }}^{2}$, or the Joule dissipation, also known as the ohmic heating, in the energy equation (see Equation (9)). Bhatti et al. [38] discovered a similar kind of response over several hybrid nanoparticles. Figure 10 shows that the Brownian motion parameter improves the temperature profile. The Brownian motion occurs as a result of the random movement of particles in the working fluid, and the collisions between these particles trigger an enhancement in the temperature field in the medium. Figure 11 shows that when the concentration of the thermophoresis parameter rises in the medium, the thermal profile rises. Since the thermophoretic force develops as a consequence of a temperature gradient, it creates a high-speed flow away from the elastic surface, causing the fluid to become more heated. As a result, the temperature profile and the thickness of the boundary layer expand. We can see that the Prandtl number opposes the rise in the temperature profile in Figure 12. Due to the fact that the thermal diffusivity considerably lowers as the Prandtl number rises, due to the decreasing temperature profile. Figure 13 shows that greater thermal slip values are in opposition to the temperature profile and the thickness of the thermal boundary layer. The thermal slip that results from Equation (11) creates a thermal leap that lowers the rate of the heat transmission between the elastic surface’s boundary regime and the wall. As a result, it tends to reduce the thermal profile, i.e., the colling influence.

### 6.3. Concentration Curves

**Figure 14:**
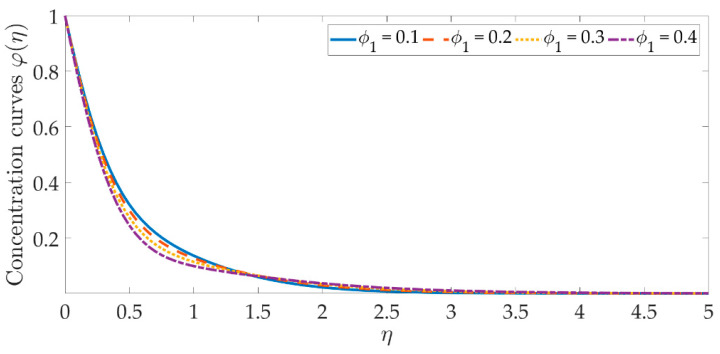
Concentration curves against the multiple values of $\phi_{1}$.

**Figure 15:**
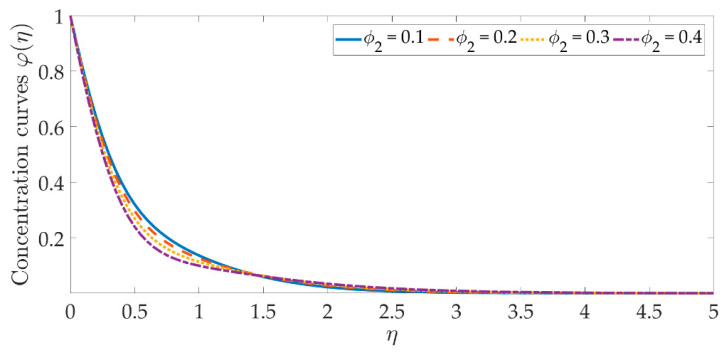
Concentration curves against the multiple values of $\phi_{2}$.

**Figure 16:**
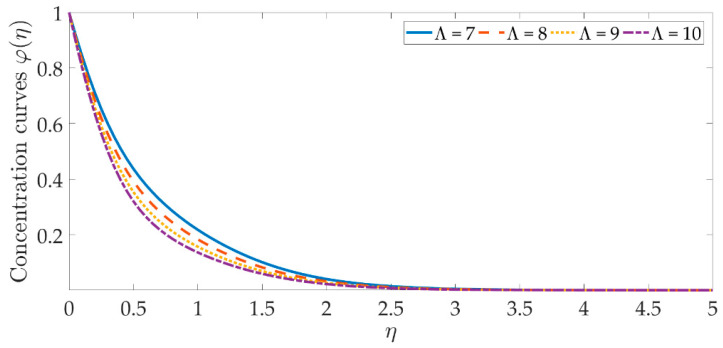
Concentration curves against the multiple values of Λ.

**Figure 17:**
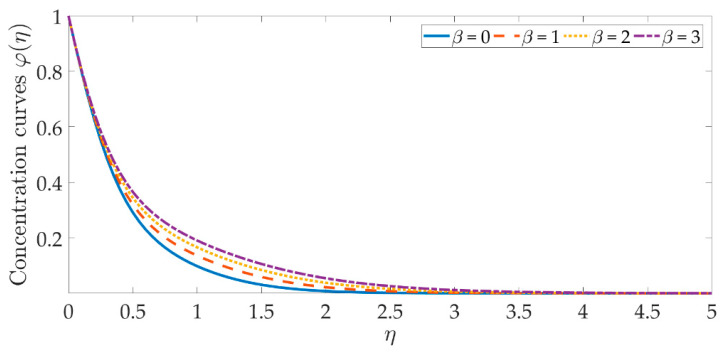
Concentration curves against the multiple values of β.

**Figure 18:**
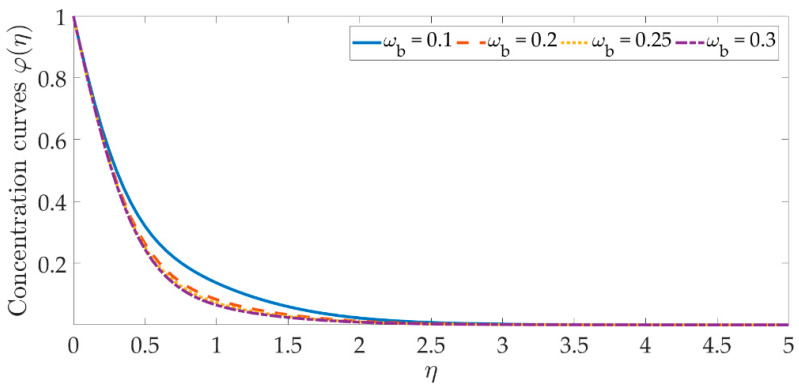
Concentration curves against the multiple values of $\omega_{b}$.

**Figure 19:**
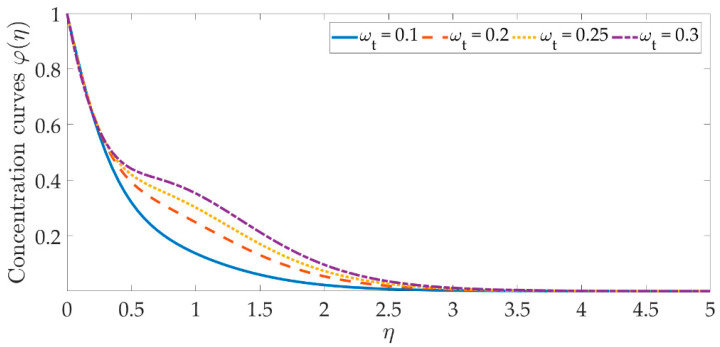
Concentration curves against the multiple values of $\omega_{t}$.

Figure 14, Figure 15, Figure 16, Figure 17, Figure 18 and Figure 19 depict the mechanism of the concentration profile, in relation to the evolving parameters for the flow modeling. The concentration profile acts similarly in the presence of both nanoparticles, as seen in Figure 14 and Figure 15. Higher concentrations of both nanoparticles diminish the concentration profile and the thickness of their respective boundary layer. When η>0, the behavior of the concentration profile reverses and exhibits a rising tendency. The trend of the Schmidt number on the concentration profile is seen in Figure 16. It is well known that nanoparticle species diffusion is impeded, resulting in a large reduction in the concentration profile and their relevant boundary layer thickness. A major influence of the Schmidt number reveals a downward trend on the concentration profile. Additionally, when the Schmidt number rises, the Brownian diffusion coefficient decreases, resulting in a decrease in the species concentration. To assess the contribution of the magnetic field on the concentration profile, Figure 17 is shown. Although the magnetic field does not directly assist the concentration equation, it is shown that the magnetic field strengthens the concentration profile. The Lorentz force indirectly affects the species diffusion field and the thickness of the boundary layer, due to the term $+f\varphi^{\prime }$ correlation with the momentum and the concentration equation. Figure 18 and Figure 19 indicate that the concentration profile exhibits a tendency and it is the opposite for both the Brownian motion and the thermophoresis force. Larger values of the thermophoresis parameter boost the concentration profile, whereas the higher values of the Brownian motion parameter oppose it. Increasing of thermophoretic force encourages the flow of nanoparticles towards the cooler area when there is a temperature gradient, which raises the concentration profile. Higher levels of the Brownian motion, to the contrary, work against the species diffusion, which lowers the concentration and thins the boundary layer.

## 7. Conclusions

A numerical analysis has been performed for the magnetized hybrid nanofluid flow across a flat elastic surface. The thermal and velocity slip boundary conditions are also considered, while analyzing the behavior of the hybrid nanofluids. The titanium oxide and copper oxide nanoparticles were explored, which are suspended in an electrically conducting and incompressible water base hybrid nanofluid. The Brownian motion, Joule heating, thermophoresis, and viscous dissipation are all considered. The numerical solutions were obtained by using the Keller–Box technique, in conjunction with a finite difference approach. Graphs and tables are used to display the findings. The following are the significant findings of this analysis:

i. The addition of hybrid nanoparticles improves the concentration, temperature, and velocity profiles, as well as the thickness of the relevant boundary layer.
ii. The conjunction of a magnetic field and velocity slip strongly opposes the fluid motion, and the maximum velocity occurs in the absence of the magnetic field and the velocity slip.
iii. The thermal profile rises as the magnetic field rises, and the similar mechanism is observed for the viscous dissipation function.
iv. Thermophoretic forces increase with the concentration and thermal profiles, as well as the thickness of the boundary layer.
v. The Brownian motion has the opposite effect on the concentration profile, when compared to the thermal profile.
vi. The increase in the Prandtl number and the thermal slip cause the thermal profile to decrease.
vii. The boundary layer thickness and the concentration profile are significantly reduced with the higher levels of the Schmidt number.
viii. The numerical comparison with the previously published findings, shows that the reported results are in perfect agreement, confirming the reliability of the presented results.

The current hybrid nanofluid flow is intriguing and deserves more investigation into the hybrid nanofluid with porous media. Furthermore, we neglected the non-Newtonian effects in the ongoing study, which may be studied in the future, using additional nanoparticles.

## Figures and Tables

**Table 1:** Thermal and physical properties of the nanofluid and hybrid nanofluid.

	Nanofluid	Hybrid Nanofluid
Dynamic viscosity	$\mu_{\;nf}=\frac{\mu_{f}}{{\left(1-\phi_{\;\mathrm{CuO}}\right)}^{2.5}}$	$\mu_{\;hnf}=\frac{\mu_{nf}}{{\left(1-\phi_{\;{\mathrm{TiO}}_{2}}\right)}^{2.5}}$
Density	$\rho_{nf}=\;\left(1-\phi_{\;1}\right)\rho_{f}+\rho_{s1}\phi_{\;1}$	$\rho_{hnf}=\left(1-\phi_{\;2}\right)\rho_{n\;f}+\;\rho_{s2}\phi_{\;2}$
Electrical conductivity	$\Omega_{nf}=\Omega_{f}\left(\frac{\Omega_{s1}\left(1+2\phi_{\;1}\right)+2\;\Omega_{f}\left(1-\phi_{\;1}\right)}{\Omega_{s1}\left(1-\phi_{\;1}\right)+\Omega_{f}\left(2+\phi_{\;1}\right)}\right)$	$\Omega_{hnf}=\Omega_{nf}\left(\frac{\Omega_{s2}\left(1+2\phi_{\;2}\right)+2\;\Omega_{f}\left(1-\phi_{\;2}\right)}{\Omega_{s2}\left(1-\phi_{\;2}\right)+\Omega_{f}\left(2+\phi_{\;2}\right)}\right)$
Thermal conductivity	$k_{nf}=k_{f}\left(\frac{2k_{f}+k_{s1}-2\left(k_{f}-k_{s1}\right)\phi_{\;1}}{2k_{f}+k_{s1}+\left(k_{f}-k_{s1}\right)\phi_{\;1}}\right)$	$k_{hnf}=k_{nf}\left(\frac{2k_{f}+k_{s2}-2\left(k_{f}-k_{s2}\right)\phi_{\;2}}{2k_{f}+k_{s2}+\left(k_{f}-k_{s2}\right)\phi_{\;2}}\right)$
Heat capacity	${\left(\rho C_{p}\right)}_{n\;f}=(1-\phi_{\;1})\;{\left(\rho \;C_{p}\right)}_{f}+\phi_{\;1}{\left(\rho \;C_{p}\right)}_{s1}$	${\left(\rho C_{p}\right)}_{hnf}=(1-\phi_{\;2})\;{\left(\rho \;C_{p}\right)}_{n\;f}+\phi_{\;2}{\left(\rho C_{p}\right)}_{s2}$

**Table 2:** Thermal and physical properties of the nanoparticles and the base fluid [36,37].

**Materials**	$\rho \left(k{g/m}^{3}\right)$	$k\left(W\;{(m\cdot K\;)}^{-1}\right)$	$\sigma \left(\Omega \;\cdot \;m^{-\;1}\right)$	$C_{p}\left(J\;{(\mathrm{kg}\cdot K\;)}^{-1}\right)$
CuO	6500	18	$5\times 10^{7}$	540
TiO_2_	4250	8.9538	$2.38\times 10^{6}$	686.6
Water (H_2_O)	997.1	0.613	0.05	4179

**Table 3:** Numerical comparison with the previous results (absence of nanoparticles) and the present results (considering $\phi_{\;1}=\phi_{\;2}=\beta =\lambda =0$).

$\omega_{\;t}$	$\omega_{\;b}$	Nusselt Number		Sherwood Number	
		Khan and Pop [37]	Present Results	Khan and Pop [37]	Present Results
0.1	0.1	0.9524	0.952871389	2.1294	2.123880810
0.2		0.6932	0.693539537	2.2740	2.275490073
0.3		0.5201	0.520233035	2.5286	2.523652658
0.1	0.2	0.5056	0.505367560	2.3819	2.378102934
	0.3	0.2522	0.250551069	2.4100	2.406859767
	0.4	0.1194	0.116936309	2.3997	2.396566472

**Table 4:** Computational results of Equation (50) using the Keller–Box method.

$\phi_{\;1}$	$\phi_{\;2}$	β	$\delta_{\;1}$	$\delta_{\;2}$	$\omega_{\;t}$	$\omega_{\;b}$	λ	χ	Λ	$E_{1}f^{\prime \prime }\left(0\right)$	$-E_{4}\theta^{\prime }\left(0\right)$	$-\varphi^{\prime }\left(0\right)$
0.1										−2.091784494	0.554205429	2.089310216
0.2										−2.764253946	0.514032226	2.142491983
0.3										−3.698417622	0.39869267	2.217793696
	0.1									−2.091784494	0.554205429	2.089310216
	0.2									−2.710048148	0.488626756	2.161265248
	0.3									−3.581149229	0.344431458	2.252125815
		0								−1.525119154	0.980466463	2.080054573
		1								−2.091784494	0.554205429	2.089310216
		2								−2.494380151	0.25088945	2.099041226
			0							−2.468615249	0.471519093	2.307729259
			0.15							−1.944277675	0.579143424	2.001485342
			0.25							−1.711542802	0.608970926	1.855244572
				0						-	0.589565233	2.0817631
				0.15						-	0.537704733	2.092641458
				0.25						-	0.50617099	2.098140057
					0.1					-	0.554205429	2.089310216
					0.2					-	0.427102644	2.212140328
					0.25					-	0.370289406	2.298776097
						0.1				-	0.554205429	2.089310216
						0.2				-	0.34058419	2.1232545
						0.25				-	0.252901943	2.125682542
							0.05			-	0.829160868	1.950498417
							0.1			-	0.554205429	2.089310216
							0.15			-	0.277032688	2.228521906
								6		-	0.557114637	2.083786479
								7		-	0.538722386	2.112309505
								8		-	0.512360152	2.142672979
									7	-	0.575111765	1.65854068
									8	-	0.566498633	1.812586477
									9	-	0.559691442	1.955498588

## Data Availability

Not applicable.
